# Social and Neurocognitive Deficits in Remitted Patients with Schizophrenia, Schizoaffective and Bipolar Disorder

**DOI:** 10.3390/healthcare9040365

**Published:** 2021-03-24

**Authors:** Liana Dehelean, Ana Maria Romosan, Bianca Oana Bucatos, Ion Papava, Rita Balint, Ana Maria Cristina Bortun, Mirela Marioara Toma, Simona Bungau, Radu Stefan Romosan

**Affiliations:** 1 Department of Neurosciences-Psychiatry, “Victor Babes” University of Medicine and Pharmacy, 300041 Timisoara, Romania; dehelean.liana@gmail.com (L.D.); ana.romosan@gmail.com (A.M.R.); bianca.bucatos@icloud.com (B.O.B.); papavaion@yahoo.com (I.P.); balint.ritka@gmail.com (R.B.); anamariacristina09@yahoo.com (A.M.C.B.); romosan.radu@gmail.com (R.S.R.); 2 Centre for Cognitive Research in Neuropsychiatric Pathology, “Victor Babes” University of Medicine and Pharmacy, 300041 Timisoara, Romania; 3 Center for Translational Research, and Systems Medicine, “Victor Babes” University of Medicine and Pharmacy, 300041 Timisoara, Romania; 4 Center for Studies in Preventive Medicine, “Victor Babes” University of Medicine and Pharmacy, 300041 Timisoara, Romania; 5 Department of Pharmacy, Faculty of Medicine and Pharmacy, University of Oradea, 410028 Oradea, Romania; mire.toma@yahoo.com

**Keywords:** neurocognitive deficits, remitted patients, cognitive function, social cognition, schizophrenia, schizoaffective, bipolar disorder

## Abstract

This study assesses the empathy level, cognitive performance and emotion recognition skills of remitted patients with schizophrenia, schizoaffective disorder and bipolar disorder, and also explores the relationship between impairments in the mentioned domains. The study was performed on 77 subjects divided into two groups: PAT sample (N = 37) included remitted patients with either schizophrenia, schizoaffective or bipolar disorder who were compared with healthy control subjects from the HC sample (N = 40). Along with sociodemographic and clinical data, empathy levels (using EQ (Empathy Quotient) scale), the ability to recognize another person’s emotional state (using RMET (Reading the Mind in the Eyes Test)), and cognitive performance (using MoCA (Montreal Cognitive Assessment) Scale) were investigated. The intensity of the psychiatric symptoms was measured with BPRS-E (Brief Psychiatric Rating Scale—Expanded). The remitted patients had lower EQ (*p* = 0.02) and RMET (*p* < 0.0001) scores than the healthy subjects. In the PAT group, RMET scores were positively correlated with MoCA total scores. Both EQ and RMET scores were negatively correlated with BPRS-E total scores. Psychiatric disorder was a significant predictor for deficits in emotion recognition. There were no significant differences in RMET, EQ and MoCA scores between patients with respect to diagnosis, the type of antipsychotic or the associated medication. In both samples, females had higher empathy levels (*p* = 0.04) and better emotion recognition abilities (*p* = 0.04) than males. Patients with schizophrenia, schizoaffective or bipolar disorder, currently in remission, displayed lower empathy levels and poorer emotion recognition skills than healthy subjects. Poor emotion recognition skills were associated with symptom severity and impairments in global cognition.

## 1. Introduction

The ability to make inferences about one’s own as well as others’ mental status is critical for effective social functioning. This is referred to as social cognition or the Theory of Mind (ToM)/mentalizing, which includes a cognitive aspect, when assuming others’ beliefs, thoughts or intentions, and an affective aspect, when inferring others’ emotions and feelings. Understanding the thoughts and feelings of other individuals (ToM) is not equivalent to empathy, which implies reacting to the observed emotions by experiencing them. Feeling what others are feeling may happen without necessarily understanding the reason why, and this is called emotional empathy. Being capable to adopt another’s perspective refers to cognitive empathy and is the equivalent of the affective aspect of ToM. In this regard, affective ToM requires the integration of cognitive ToM with empathy [1]. Sympathy appears when responding to others’ emotions in a supportive way [2,3].

As mentioned above, inferring one’s own or others’ mental status requires an adequate cognitive functioning. This implies the capacity to focus attention, to structure perception, to retrieve information from episodic and semantic memory, to associate data through working memory, to simulate, to create representations and meta-representations, to use language and to make predictions using executive functions. The bottom-up processes involved in static and movement perception interact with the top-down processes, which are required for meta-representations, executive functions or language. In this respect, two other components of ToM are described: social perceptive and social cognitive. The social perceptive dimension is involved in understanding others’ mental status through mind simulation, without the use of mental representations (simulation theory). The social cognitive dimension helps the individual to understanding others’ mental status through explicit mental representations and language [3,4,5,6].

The ability to recognize others’ mental states develops through childhood and adolescence and requires social interactions. It is detectable at 18 months of age, firmly establishes at 4–6 years and is fully developed in young adulthood. Adolescence is critical for building up the social perceptive component of ToM [4]. Age-related decline in executive abilities and episodic memory may explain the lower performance of ToM tasks in elderly subjects [5].

The core neural structures involved in ToM are as follows: the medial prefrontal cortex (mPFC), the anterior temporal pole, the temporal–parietal junction (TPJ), and the precuneus. Some brain structures build cognitive ToM networks (dorsal mPFC, dorsal anterior cingulated cortex, dorsal striatum) while others build affective ToM networks (ventral mPFC, orbital frontal cortex, ventral anterior cingulated cortex, amygdala, and ventral striatum) [6]. In addition, certain brain structures are involved in interpersonal processing, while others are involved in intrapersonal (self-referential) processing. The mentalizing process consists of internal simulations made when disconnected from the external environment: creating mental imageries, reflecting about past experiences, predicting the future or projecting oneself in other people’s mind (i.e., adopting other people’s points of view, taking perspective). In this process, the default mode network (DMN) is active. The main DMN structures include the mPFC, medial temporal cortex (hippocampus), angular gyrus (part of TPJ), posterior cingulated cortex (PCC) and precuneus. The DMN uses the mirror neurons system. This is formed by neurons of the inferior frontal gyrus (IFG) and the inferior parietal lobule (IPL) connected through the superior temporal sulcus (STS) with the areas involved in face perception. Mirror neurons are active when executing an action or observing another person performing the same action. The TPJ and inferior parietal regions are involved in mental processing distinguishing between the self’s and other people’s perspectives [7].

In this respect, a common network appears to be involved in both autobiographical (episodic) memory and mentalizing (ToM). This fact supports the hypothesis that autobiographical (episodic) memory, a function of the hippocampus, is required in ToM. The network is made of midline structures such as the mPFC, precuneus and PCC (mental imagery, retrieval of episodic memory), medial temporal lobe (MTL) and hippocampus (autobiographic episodic memory). These midline structures mediate the “familiar” ToM, when inferring about the mental status of a known person. The “unfamiliar” ToM, when inferring about the mental status of an unknown person, relies on the connections made by lateral structures such as the anterior temporal pole (semantic memory) and TPJ (language processing, attention) with ToM structures. According to Moreau and collaborators, ToM deficits observed in patients with dementia in Alzheimer’s disease may be the result of poor memory retrieval of past social interactions [8]. Aging is associated with a decreased connectivity in the DMN [9], more specifically between mPFC and PCC [10]. In the early stages of Alzheimer’s disease dementia, connectivity between DMN neurons is further affected by alterations in the structure and functionality of the hippocampus, precuneus, and PCC [8,11,12,13,14,15].

In conclusion, the interpersonal cognitive ToM relies on circuits involving the dorsolateral prefrontal cortex, dorsal mPFC, dorsal anterior cingulated cortex, superior temporal sulcus and TPJ. Interpersonal affective ToM involves circuits formed by the IFG, orbital frontal cortex, and ventral mPFC. Intrapersonal ToM is based on circuits involving the precuneus, PCC, middle cingulated cortex, ventral anterior cingulated cortex, and ventral mPFC [2].

Impaired abilities to process others’ mental states, together with cognitive dysfunctions, are frequently described in patients with schizophrenia, schizoaffective disorder and bipolar disorder [16,17,18,19,20,21]. These deficits manifest themselves during acute episodes, as well as in remission. They have a negative impact on patients’ social rehabilitation, which can result in social isolation and in diminishing quality of life [22].

The hypothesis considering poor ToM performance as a trait deficit is that ToM performance is worse in remitted patients with schizophrenia and schizoaffective disorder than in their first-degree relatives and these relatives perform worse than healthy controls [23]. Bora and collaborators suggest that ToM dysfunctions may represent a reflection of subclinical symptoms rather than a real trait abnormality [18].

ToM performance was found to be impaired in childhood onset schizophrenia [24], but an earlier onset of the disorder may affect the development of ToM. Li and collaborators found no correlation between ToM and duration of the disorder in adolescent onset schizophrenia [25]. ToM impairment was associated with an earlier age of illness onset in bipolar pediatric patients [26].

The study has three main objectives. The first one is to assess empathy levels, emotion recognition abilities and cognitive performance of remitted patients with schizophrenia, schizoaffective disorder, and bipolar disorder (with at least 2 episodes with psychotic features), taking into account several potential influential factors (e.g., age at study entry, gender, educational level, professional status, smoking habits and alcohol misuse). Based on prior studies in the literature, we expect to find considerable deficits in the aforementioned domains when comparing patients with chronic psychosis (the PAT sample) and a healthy control group (HC). The second objective is to examine the influence of affective ToM performance and empathy on these patients with psychosis. We presumed that lower empathy levels and diminished social cognition may constitute possible markers for the existence of a psychiatric disorder from the psychotic spectrum. The third objective is to explore the relationship between empathy, affective ToM abilities and cognitive impairment in these patients. 

## 2. Materials and Methods

### 2.1. Participants Selection

The study is cross-sectional and included 37 outpatients (PAT sample) with either schizophrenia, schizoaffective disorder or bipolar disorder diagnosed by psychiatrists (with at least two episodes with psychotic features in the patient history), currently in remission, according to ICD-10 diagnostic criteria; and 40 healthy subjects (HC sample, which showed normal values for BPRS-E and MOCA), matched for age and gender. The research was carried out between January and May 2019 on patients registered at the “Pius Brinzeu” County Clinic Emergency Hospital, Timisoara, Romania. The exclusion criteria for the PAT sample are as follows: patients in the acute phase of the disorder, personal history of organic mental disorders, personal history of psychoactive substance use (other than alcohol and tobacco).

### 2.2. Ethical Statement

After being informed about the aim of the study, each participant signed a written informed consent prior to their inclusion in the study. Research was conducted in agreement with Helsinki Declaration guidelines for experiments involving human beings and it was also approved by the Scientific Research Ethics Committee for Scientific Research of the County Clinic Emergency Hospital “Pius Brinzeu”, Timisoara, Romania (Reference number 157/2019).

### 2.3. Measurements

Sociodemographic data (age at inclusion in the study period, gender, level of education, professional status) and clinical data (current psychiatric treatment, tobacco and alcohol misuse) were collected for each participant.

Performance at ToM tests is measured through visual (static or dynamic) and verbal stimuli. Some of the tests assess affective ToM (pictorial or verbal narratives describing somebody’s emotions) while others measure cognitive ToM using tasks that require understanding of sarcasm, deception, social faux pas or false beliefs. The dynamic–visual way of presenting the stimuli is richer in information than the static–visual way (pictures of people’s eyes or cartoons) and the verbal way (verbal narratives describing situations). However, verbally presented tests are less affected by age than visual measures [27]. To minimize the age effect on cognitive functions, we used multiple channel stimuli: verbal (EQ) and static–visual (RMET).

The EQ (Empathy Quotient) [3] is a self-administered questionnaire used to measure empathy in adults with normal intelligence. Being a subjective evaluation of one’s own level of empathy, it may not necessarily reflect the reality. It has 60 items among which 40 are related to empathy and 20 are control items. Each item consists of a first-person statement which may be rated from “strongly agree”, “slightly agree”, “slightly disagree”, or “strongly disagree”. The maximum score is 80.

The revised version of RMET (Reading the Mind in the Eyes Test) [28] consists of 36 photographs depicting four possible emotions (“suspicious”, “ashamed”, “frightened”, and “serious”) that correspond to four words printed on each photograph. The subject performing the test has to choose the word that best describes the depicted person’s mental state by analyzing the eye region. The score is the sum of the correct answers of each subject. The maximum score is 36.

Cognitive functioning was assessed with MoCA (Montreal Cognitive Assessment) [29]. The test has 30 items assessing six cognitive domains such as executive functions (EF), visuospatial abilities (VSA), language (LG), attention (AT), memory (MEM), and orientation (OT). Scores below 26 are considered abnormal.

The presence and severity of psychiatric symptoms were assessed with the BPRS-E (Brief Psychiatric Rating Scale—Expanded, version 4.0). The scale was performed by a trained psychiatrist using the BPRS-E administration manual. The 24-item BPRS-E was developed by Lukoff, Nuechterlein and Ventura [30] from the previous 18 item BPRS introduced by Overall and Gorham [31]. The use of BPRS-E was preferred in this study because it seems to better cover the schizophrenic and affective symptoms than the 18-item BPRS [32]. Symptoms are rated on a 7-point Likert scale with a maximum score of 168. The psychiatric symptoms may be grouped in 4 or 5 clusters of symptoms [32,33,34]. All measurements were performed when the patients achieved the remission state.

### 2.4. Statistical Analysis

Statistical analysis was computed using IBM SPSS Statistics (version 20; IBM Corporation, Armonk, NY, USA). As data showed a non-Gaussian distribution (tested using the Shapiro–Wilk normality test), differences between the patient group and the HC group were checked using non-parametric tests (the Mann–Whitney U test with the Bonferroni correction), while the χ^2^ (chi-square) test was performed in order to examine differences between categorical data. Potential associations between scale scores were assessed using Spearman’s correlation coefficient. The level of significance was set at 0.05 and all the results were two-tailed. The relationship between the psychiatric disorder and RMET and EQ scores was analyzed using a logistic regression model, for which values were reported such as: χ^2^ statistics, percentage of correct predictions, coefficient estimates (β) and Wald statistics. The presence of the psychiatric condition was introduced as the dependent variable (DV), where HC participants were coded as 0, and the patient sample was coded as 1. RMET and EQ scores, together with participants’ gender, age and educational level, were considered the independent variables (IVs) of the regression model.

## 3. Results

The demographic and clinical characteristics of both samples are presented in Table 1 and Table 2. Subjects in the PAT sample were in remission and displaying mild cognitive impairment, as illustrated in Table 2 and Table 3.

EQ, RMET, MoCA, and BPRS-E scores were analyzed with respect to sociodemographic and clinical parameters (age at study entry, gender, level of education, professional status, smoking, and alcohol misuse).

Age at study entry was negatively correlated with RMET score (rs = −0.4, *p* < 0.0001), MoCA total score (rs = −0.35, *p* = 0.03) and MoCA score for visuospatial abilities (rs = −0.33, *p* = 0.04). No correlation was found between BPRS-E and age at study entry.

The level of education had a significant impact only on RMET score (H = 23.07, *p* < 0.0001), subjects with more than 12 years of education having higher scores than those with medium level of education (*p* < 0.0001), and also than those with a lower level of education (*p* = 0.04). No differences were found between subjects with medium level and lower levels of education. The EQ, MoCA and BPRS-E scores were not influenced by the level of education. In the PAT sample, RMET scores were significantly higher (U = 200, Z = −4.16, *p* < 0.0001) and BPRS-E scores significantly lower (U = 72, Z = −3.01, *p* = 0.003) in employed patients than in those unemployed. EQ and MoCA scores were not influenced by professional status.

Female subjects from both samples had higher scores for empathy (U = 480.5, Z = −2.08, *p* = 0.04), and better abilities in recognizing emotions (U = 479, Z = −2.09, *p* = 0.04) than males. No differences between males and females were found regarding the MoCA score and BPRS-E score.

Alcohol misuse and smoking did not significantly influence the scores for EQ, RMET, MoCA, or BPRS-E.

We found no significant differences in EQ, RMET, MoCA and BPRS-E scores between patients with respect to the type of the psychiatric disorder (schizophrenia, schizoaffective disorder, bipolar disorder), the type of antipsychotic (aripiprazole, clozapine, olanzapine, paliperidone, quetiapine, risperidone), or the associated medication (mood stabilizer or antidepressant).

The regression model testing the relationship between the psychiatric disorder and RMET and EQ scores was statistically significant (χ^2^ = 27.74, *p* < 0.0001), explaining 40% of the variance in the case of presence of the disorder and correctly classifying 75.3% of cases. Lower RMET scores were associated with a higher probability for the existence of a psychiatric disorder from the psychotic spectrum (β = 0.25, Wald = 11.34, *p* = 0.001), while EQ scores (β = 0.02, Wald = 0.56, *p* = 0.45), age (β = 0.03, Wald = 1.53, *p* = 0.21), education (β = 0.39, Wald = 0.42, *p* = 0.51) and gender (β = 0.22, Wald = 0.13, *p* = 0.71) did not contribute significantly to the model.

The associations between empathy, emotion recognition skills and cognitive performance in the PAT sample are depicted in Table 4.

## 4. Discussion

Patients with schizophrenia, schizoaffective disorder or bipolar disorder, currently in remission, had lower empathy levels and poorer skills in recognizing emotions than healthy controls. Fewer of them achieved a higher level of education (>12 years) compared with the healthy subjects. Additionally, unemployment was also associated with lower RMET scores. Both EQ and RMET were negatively correlated with BPRS-E total scores. In this respect, the disorder itself, a poorer quality of remission, lower levels of education and unemployment all accounted for the lower ability to recognize emotions in the PAT sample.

Regarding professional status, employed patients had lower BPRS-E scores reflecting a better quality of remission. Their higher RMET score may represent evidence for better skills of emotion recognition due to a better remission and/or their involvement in social activities at work.

Age at study entry was negatively correlated with RMET score, MoCA total score and MoCA subscore for visuospatial abilities. Older age can be associated with reduced interest or involvement in social activities and a decline in visuospatial skills. These results are in accordance with similar findings about the effect of age on ToM performance or RMET scores [5].

Female subjects showed higher empathy levels and better emotion recognition abilities than males. This result replicates other studies about women performing better than men on ToM tests.

Lower RMET scores were associated with a higher probability of the existence of a psychiatric disorder from the analyzed schizophrenic and affective spectrum. This is in agreement with the finding that social cognition in schizophrenia is a significantly better predictor for the probability of being a patient than nonsocial cognition [35].

Positive correlations were found in the PAT group between affective ToM performance and overall cognitive performance, but also between RMET scores and MoCA attention subscore. These findings suggest an association between emotion recognition skills and cognitive performance, mainly the capacity to concentrate attention. The negative correlations between BPRS-E score and MoCA total score, MoCA attention subscore and MoCA memory subscore may reflect the association between mild psychiatric symptoms and cognitive impairments, especially regarding domains such as attention and memory.

Both EQ and RMET scores were negatively correlated with BPRS-E total scores, suggesting that lower empathy levels and lower emotion recognition abilities coexist with mild psychiatric symptoms. According to Harrington, the defective processing of others’ thoughts and intentions may result in persecutory and reference delusions [36]. Abu-Akel and Bailey suggest that patients with schizophrenia presenting positive symptoms do not lack an understanding of others’ mental state but over-attribute mental states to others [37]. Schenkel and collaborators suggest that ToM deficits in schizophrenia may be responsible for poor premorbid social functioning and more disorganized symptoms after disorder onset [38]. A meta-analysis by Sprong and collaborators found that patients with schizophrenia having disorganization symptoms performed worse on mentalizing tasks (ToM) than those with other symptoms (i.e., paranoid symptoms). The subjects in remission also displayed significant impairment, suggesting that ToM deficits may be trait-dependent [39].

Our results also suggest that the effect of the disorder on emotion recognition skills may not be the result of existing executive dysfunctions, as we found no correlations between RMET and MoCA scores for executive functions. This fact supports the findings of Ahmed and Miller, reporting that executive dysfunctions do not significantly affect RMET scores [40]. The lower performance in emotion recognition could be the result of attention impairments, as a positive correlation was found between MoCA attention subscore and RMET score. This correlation suggests an association between attention and the ability to recognize emotions in another person. Yet, the association between attention and emotion recognition is bidirectional. Studies conducted on inpatients [41] and remitted patients [42,43] with schizophrenia revealed the existence of abnormalities in both attention and emotion recognition. Attention shaping or redirecting visual attention to relevant features of faces exhibiting emotions improves the recognition of emotions in these patients. Dadds and collaborators found that the deficit in fear recognition of children and adolescents with psychopathy may be driven by the visual neglect of the eye region of other people’s faces and can be temporarily reversed by focusing attention to this region [44]. Begeer and collaborators revealed that in neutral conditions, high functioning children with autism pay less attention to facial emotions compared to the control group, but they may increase attention when asked to focus on the behavioral outcomes of the presented emotions [45]. In patients with ADHD, the literature data show contradictory results ranging from implicating visual attention deficits in the impaired facial emotion recognition in boys with ADHD [46] to no association of ADHD with deficits in emotion recognition [47] or little evidence of deficits in social cognition [48].

The negative correlations found between BPRS-E scores and MoCA scores (total, attention and memory) suggest that the presence of mild psychiatric symptoms coexists with cognitive impairments regarding attention and memory. The literature data show that while positive psychotic symptoms such as hallucinations and delusions have little or no association with cognitive dysfunctions, negative and disorganized symptoms show a stronger relationship with discrete cognitive impairments such as immediate recall, attention, verbal and non-verbal memory, verbal IQ and full-scale IQ [49,50,51,52,53].

However, the main limitations of our study are that the PAT and HC samples were not matched to the educational level and that the number of subjects included in the study was not very high. Another possible limitation of the study may be the fact that the patients were not enrolled in any psychotherapeutic interventions, which might have had some influence on the neurocognitive deficits.

## 5. Conclusions

Patients with schizophrenia, schizoaffective disorder or bipolar disorder, currently in remission, displayed lower empathy levels and poorer emotion recognition skills than healthy subjects. The results were not influenced by the psychiatric diagnosis, the type of antipsychotic or of the adjunctive medication. Psychiatric disorder was a predictor for the deficits in emotion recognition. Moreover, poor emotion recognition skills were associated with symptom severity and impairments in global cognition.

## Figures and Tables

**Table 1:** Sample demographic characteristics.

Demographic Characteristics		PAT		HC		^1^ PAT vs. HC	*p*-Value
		N = 37	%	N = 40	%		
Gender	Males	16	43.2	11	27.5	χ^2^: 2.09	0.15
	Females	21	56.8	29	72.5		
Age (years)	M, SD	41.65	9.23	39.02	15.7	U = 554; Z = −1.89	0.06
Years of education	1–8	4	10.8	0	0	χ^2^: 7.03	0.03
	9–12	24	37.8	10	25		
	>12	19	51.4	30	75		
Professional status	Employed	18	48.6	40	100	χ^2^: 27.26	<0.0001
	Unemployed	19	51.4	0	0		

Legend: PAT, patients; HC, healthy controls; M, mean; SD, standard deviation; ^1^ Chi square test (χ^2^) or Mann–Whitney U test.

**Table 2:** Sample clinical characteristics.

Sample Clinical Characteristics		PAT		HC		^1^ PAT vs. HC	*p*-Value
		N = 37	%	N = 40	%		
Diagnosis	Schizophrenia	26	70.3	-	-	-	-
	Schizoaffective disorder	6	16.2				
	Bipolar disorder	5	13.5				
Antipsychotic treatment	Aripiprazole	6	16.2	-	-	-	-
	Clozapine	5	13.5				
	Olanzapine	14	37.9				
	Paliperidone	5	13.5				
	Quetiapine	3	8.1				
	Risperidone	4	10.8				
Mood stabilizer or antidepressant	Yes	20	54.1	-	-	-	-
	No	17	45.9				
Alcohol misuse	Yes	5	13.5	0	0	χ^2^: 5.78	0.02
	No	32	86.5	40	100		
Smoking	Yes	10	27.1	9	22.5	χ^2^: 0.21	0.65
	No	27	72.9	31	77.5		
RMET correct answers, SD		19.27	5.22	25.3	4.31	U:274, Z = −4.76	<0.0001
EQ total score, SD		37.43	10.34	43.07	10.81	U:508, Z = −2.36	0.02
BPRS-E total score, SD		35.54	9.83	24.55	1.03	U:91, Z = −7.36	<0.0001

Legend: PAT, patients; HC, healthy controls; M, mean; SD, standard deviation; RMET, Reading the Mind in the Eyes Test; EQ, Empathy Quotient; BPRS-E, Brief Psychiatry Rating Scale—Expanded, ^1^ Chi square test (χ^2^) or Mann–Whitney U test.

**Table 3:** MoCA scores of the PAT sample.

MoCA	PAT: Mean Scores (SD)	HC: Mean Scores (SD)	Mann–Whitney U Test U; Z	*p*-Value
Total	25.16 (3.61)	29.69 (0.51)	111; −7.22	<0.0001
Executive functions (EF)	2.54 (1.28)	3.03 (0.25)	317; −5.94	<0.0001
Visuospatial abilities (VSA)	3.41 (0.83)	3.95 (0.21)	553; −4.15	<0.0001
Language (LG)	4.43 (0.6)	5 (0)	430; −5.45	<0.0001
Memory (MEM)	3.45 (1.26)	4.95 (0.21)	237; −6.64	<0.0001
Attention (AT)	5.21 (1.08)	5.88 (0.32)	520; −4.12	<0.0001
Orientation (OT)	5.89 (0.65)	5.97 (0.15)	902; −0.03	0.97

Legend: PAT, patients; HC, healthy controls; MoCA, Montreal Cognitive Assessment; SD, standard deviation.

**Table 4:** Correlation coefficients and their statistical significance for RMET, EQ, BPRS and MoCA scores for the PAT sample.

Correl. Coef.	1	2	3	4	5	6	7	8	9	10
**1**	1	-	-	-	-	-	-	-	-	-
**2**	0.94 (0.058)	1	-	-	-	-	-	-	-	-
**3**	−0.46 (0.005 **)	−0.38 (0.02 *)	1	-	-	-	-	-	-	-
**4**	0.35 (0.03 *)	−0.02 (0.91)	−0.37 (0.02 *)	1	-	-	-	-	-	-
**5**	0.24 (0.15)	−0.16 (0.33)	−0.09 (0.60)	0.78 (<0.0001 **)	1	-	-	-	-	-
**6**	0.20 (0.22)	0.10 (0.54)	−0.18 (0.29)	0.67 (<0.0001 **)	0.58 (<0.0001 ***)	1	-	-	-	-
**7**	0.17 (0.31)	−0.12 (0.46)	−0.10 (0.55)	0.39 (0.02 *)	0.11 (0.46)	0.29 (0.07)	1	-	-	-
**8**	0.18 (0.28)	0.21 (0.21)	−0.36 (0.03 *)	0.78 (<0.0001 **)	0.52 (0.001 **)	0.42 (0.01 *)	0.15 (0.37)	1	-	-
**9**	0.40 (0.01 *)	0.16 (0.33)	−0.49 (0.002 **)	0.32 (0.04 *)	0.07 (0.68)	−0.02 (0.86)	0.06 (0.73)	0.32 (0.05)	1	-
**10**	0.21 (0.19)	−0.16 (0.33)	−0.25 (0.13)	0.28 (0.09)	0.20 (0.23)	0.31 (0.06)	−0.16 (0.32)	0.29 (0.08)	0.30 (0.07)	1

Legend: **1**—RMET, Reading the Mind in the Eyes Test; **2**—EQ, Empathy Quotient; **3**—BPRS-E, Brief Psychiatry Rating Scale-Expanded; MoCA, Montreal Cognitive Assessment; **4**—MoCA total; **5**—MoCA executive; **6**—MocA visuo-spatial; **7**—MoCA language; **8**—MoCA memory; **9**—MoCA attention; **10**—MoCA orientation. * Correlation is significant at the 0.05 level (2-tailed); ** Correlation is significant at the 0.01 level (2-tailed). *** Correlation is significant at the 0.001 level (2-tailed).

## Data Availability

Data are available in the database of the hospital where the study was performed, namely “Pius Brinzeu” County Clinic Emergency Hospital.
